# Left Main Revascularization in Patients with Chronic Kidney Disease: A Systematic Review and Meta-Analysis

**DOI:** 10.2174/011573403X396917250910215747

**Published:** 2025-09-17

**Authors:** Ioannis Gialamas, Konstantinos Kalogeras, Panteleimon Pantelidis, Georgios E. Zakynthinos, Antonios Lysandrou, Efstratios Katsianos, Athina Goliopoulou, Maria Ioanna Gounaridi, Nikolaos Vythoulkas-Biotis, Ourania Katsarou, Evangelos Oikonomou, Gerasimos Siasos, Manolis Vavuranakis

**Affiliations:** 1 3^rd^ Department of Cardiology, Sotiria Chest Disease Hospital, Medical School, National and Kapodistrian University of Athens, Athens, Greece;; 2 Cardiovascular Division of Brigham and Women's Hospital, Harvard Medical School, Boston, USA

**Keywords:** Left main coronary artery disease, chronic kidney disease, revascularization, systematic review and meta-analysis, percutaneous coronary intervention, coronary artery bypass grafting

## Abstract

**Introduction/Objective:**

This systematic review and meta-analysis compares percutaneous coronary intervention (PCI) with coronary artery bypass grafting (CABG) as revascularization strategies for patients with left main coronary artery disease (LMCAD) and chronic kidney disease (CKD).

**Methods:**

A comprehensive search of PubMed, Embase, and CENTRAL was conducted, with a pre-registered study protocol registered on PROSPERO (ID: CRD42024496529). The primary endpoint was major adverse cardiac and cerebrovascular events (MACCE), a composite of all-cause mortality, myocardial infarction (MI), stroke, or ischemia-driven revascularization. Secondary endpoints included each component of MACCE and 30-day all-cause mortality.

**Results:**

Seven studies were analyzed, including five cohort studies and two subanalyses of randomized clinical trials, encompassing 3,376 patients. PCI was associated with a higher incidence of MACCE (hazard ratio [HR]: 1.50; 95% confidence interval [CI] 1.26-1.78), driven by all-cause mortality (HR: 1.38; 95% CI 1.07-1.79), MI (HR: 1.76; 95% CI 1.15-2.71), and ischemia-driven revascularization (HR: 3.66; 95% CI 1.84-7.30). There were no differences in stroke rates (HR: 0.70; 95% CI 0.40-1.22) or 30-day all-cause mortality (odds ratio [OR]: 1.28; 95% CI 0.85-1.94).

**Discussion:**

While previous studies have reported conflicting evidence regarding the non-inferiority of PCI to CABG in patients with LMCAD, our pooled analysis demonstrates an increased incidence of MACCE in the PCI group, primarily driven by higher rates of all-cause mortality, MI, and ischemia-driven revascularization. The findings suggest that CKD may play a role in clinical outcomes comparable to diabetes in multivessel disease and should be a key factor in revascularization decisions.

**Conclusion:**

CABG is associated with superior long-term outcomes compared to PCI in patients with LMCAD and CKD. However, dedicated randomized controlled trials stratified by CKD stage are essential to guide optimal treatment strategies in this high-risk population.

## 1 INTRODUCTION

The prognosis of patients with left main coronary artery disease (LMCAD) remains poor without intervention, with a reported three-year survival rate of only 37% [1, 2]. A landmark meta-analysis from the 1990s demonstrated that coronary artery bypass grafting (CABG) reduces the mortality of these patients by approximately 75%, establishing it as the standard of care for decades [3]. In recent years, percutaneous coronary intervention (PCI) has emerged as a less invasive alternative, particularly for high surgical risk patients. However, the evidence supporting the non-inferiority of PCI to CABG in patients with LMCAD remains inconclusive, with major trials yielding conflicting results [4-7].

Chronic kidney disease (CKD) is a prevalent comorbidity in patients with LMCAD and is pathophysiologically linked to accelerated atherosclerosis, systemic inflammation, endothelial dysfunction, and increased thrombogenicity, all of which contribute to the progression of coronary artery disease [8-11]. CKD not only exacerbates cardiovascular risk but also complicates the management of LMCAD by increasing the risks of contrast-induced kidney injury during PCI and both bleeding and thrombosis in CABG, thereby influencing the choice and outcomes of revascularization strategies [11]. Despite the relatively high prevalence of patients with both LMCAD and CKD [12], there is currently no evidence-based guidance on the optimal revascularization approach for this specific high-risk population. This is largely due to the underrepresentation of CKD patients in major trials comparing PCI and CABG, as well as the lack of stratified outcome analyses in existing meta-analyses, leaving a significant gap in the evidence base.

This systematic review and meta-analysis aims to bridge the evidence gap by comparing the short- and long-term outcomes of PCI and CABG in patients with concomitant LMCAD and CKD, providing critical insights that can help to optimize treatment strategies for this high-risk population.

## 2 METHODS

The methodology of this systematic review and meta-analysis followed the “Preferred Reporting Items for Systematic Reviews and Meta-Analyses (PRISMA) Statement and Recommendations” and the principles of the “Cochrane Handbook for Systematic Reviews” [13, 14]. The prespecified protocol was registered in the PROSPERO database (ID: CRD42024496529).

### 2.1 Search Strategy

Two independent researchers conducted a systematic search across three major databases-MEDLINE (*via* PubMed), Embase, and CENTRAL (*via* Cochrane)-from their inception through February 2025. The search strategy incorporated alternative spellings, truncations, and variations of terms related to “percutaneous coronary intervention”, “coronary artery bypass graft surgery”, “left main coronary artery disease”, and “chronic kidney disease” (see Supplementary Material). Additionally, a snowballing approach was employed by screening the reference lists of included studies.

### 2.2 Study Selection and Eligibility Criteria

Studies were eligible if they enrolled patients with both LMCAD, defined as >50% stenosis on angiography, and chronic kidney disease (CKD), defined as a glomerular filtration rate (GFR) <60 mL/min/1.73 m^2^ according to the “Kidney Disease Improving Global Outcomes (KDIGO)” guidelines [15]. To ensure consistency in CKD classification, eligible studies were required to assess renal function using GFR and not serum creatinine levels. The studies also had to compare PCI with CABG and report clinical outcomes of interest with sufficient data to extract hazard ratios or equivalent measures. Both observational studies and randomized controlled trials published in English as full-text, peer-reviewed articles were considered (see Supplementary Material). Grey literature and conference abstracts were excluded from our systematic review to prioritize peer-reviewed publications that have undergone full methodological scrutiny.

### 2.3 Quality Assessment

The risk of bias in all included studies was evaluated by two reviewers using the “Risk of Bias 2 (RoB2)” tool for randomized trials and the “Risk Of Bias In Non-randomized Studies - of Interventions (ROBINS-I)” for non-randomized studies [16]. For RoB2, we evaluated five domains: randomization process, deviations from intended interventions, missing outcome data, outcome measurement, and selective reporting. Each study was judged as having ‘low,’ ‘some concerns,’ or ‘high’ risk of bias. For ROBINS-I, we assessed bias across seven domains (*e.g.*, confounding, participant selection, classification of interventions) and categorized studies as ‘low,’ ‘moderate,’ ‘serious,’ or ‘critical’ risk of bias. Any discrepancies were resolved through consensus. Publication bias assessment using Egger’s test was limited to the primary outcome, due to the small number of studies assessed for secondary outcomes.

### 2.4 Data Extraction and Outcomes

The data from the eligible studies were extracted by two independent researchers using a predefined spreadsheet form. The extracted data included information on the study design, the characteristics of the patients, and the outcomes of interest (see Supplementary Material). Continuous variables were extracted as means and standard deviations (SD), while categorical variables were recorded as counts. The reported medians and interquartile ranges (IQR) were transformed into means and SD using the Wan and Luo formulas when necessary [17, 18]. Hazard ratios (HR) for the preferred outcomes were extracted from Cox models incorporating time into the analysis, in anticipation of a considerable number of events. In instances where hazard ratios were not available but survival curves were provided, we used the WebPlotDigitizer software and the methodology described by Tierney *et al*. [19, 20]. The number of occurrences was extracted to analyze the 30-day all-cause mortality. When available, the matched data were extracted from cohort studies. Any discrepancies were resolved through discussion until a consensus was reached. Authors of selected studies were contacted to obtain any missing data relevant to the analysis, when necessary.

The primary outcome of interest was “major adverse cardiac and cerebrovascular events” (MACCE), defined as the composite of all-cause mortality, myocardial infarction (MI), stroke, or any ischemia-driven revascularization. Secondary outcomes of interest included individual components of MACCE (all-cause mortality, MI, stroke, and any ischemia-driven revascularization), cardiovascular death, acute kidney injury events, and the classical 3-point MACCE, defined as the composite of all-cause mortality, myocardial infarction, or stroke (see Supplementary Material).

### 2.5 Statistical Analysis

Statistical analyses were performed in R software (version 3.6.3). For the meta-analysis, crude HRs from at least three studies were pooled, with a significance threshold set at *p <* 0.05. The decision to utilize crude instead of adjusted HRs was made to maintain consistency across the included studies and to avoid introducing bias from heterogeneous adjustment models. Heterogeneity across studies was evaluated using the I^2^ statistic, with values greater than 50% indicating substantial heterogeneity. Due to variability in clinical settings, patient populations, CKD stages, and study designs, a random effects model was applied. To assess the robustness of the outcomes and to explore potential sources of heterogeneity, subgroup analyses were conducted based on randomization type, CKD stage, risk of bias assessment, and patient age (using a cut-off of 70 years). Publication bias was assessed using Egger's test for the primary outcome of MACCE.

## 3 RESULTS

### 3.1 Search Results

**Fig. (1):**
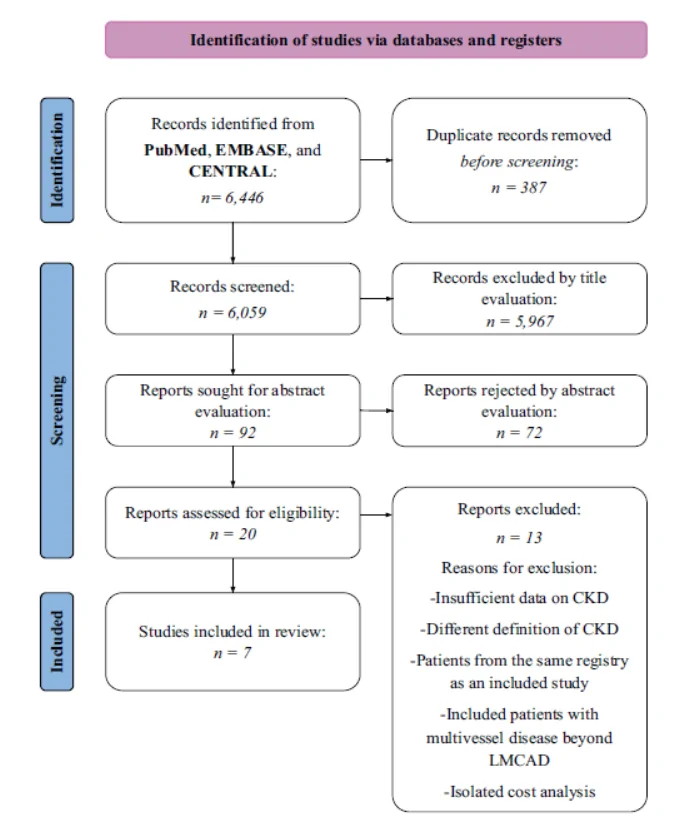
PRISMA flow chart illustrating the search progress.

The selection process is illustrated in the PRISMA flow diagram (Fig. **1**). Out of a total of 6,059 records screened, 7 studies met the inclusion criteria and were included in the final analysis [21-27]. Efforts were made to contact the corresponding authors of 3 studies, however, no response was received [28-30]. Two studies used the same database of patients, therefore, the most appropriate study was chosen [21, 31]. Further details regarding the search results and reasons for exclusion are provided in the Supplementary Material. The raw data for all outcomes are included in Tables **S1-S3**.

### 3.2 Study Characteristics and Risk of Bias

The included studies comprised a total of 3,376 patients with both LMCAD and CKD, who underwent either PCI (*n*=1,634) or CABG (*n*=1,742), with an average follow-up time of 3.7 years [21, 23, 25-27]. The studies consisted of five cohort studies [21, 23, 25-27], along with two subanalyses from randomized controlled trials (RCTs) [22, 24], conducted across various regions: one international study [22], 4 from Asia [21, 23, 25, 26], 2 from the United States [24, 27], with none exclusively from Europe (see Table **1**).

The baseline characteristics of patients undergoing PCI and CABG were largely comparable (Table **1**). The weighted mean age was 70.4 years in the PCI group and 68.6 years in the CABG group, with male patients comprising 75.3% and 76.5% of each group, respectively. Comorbidities-including hypertension, dyslipidemia, smoking, and diabetes mellitus-were similarly prevalent between the groups. Left ventricular ejection fraction (LVEF), reported across four studies, ranged from 45% ± 12% to 62% ± 12% in PCI patients and from 46% ± 13% to 57% ± 12% in CABG patients. Renal function, as measured by estimated glomerular filtration rate (eGFR), was also comparable, with weighted means of 44 mL/min/1.73 m^2^ for PCI and 43 mL/min/1.73 m^2^ for CABG. The primary distinction between the groups was less complex coronary anatomy in the PCI group (see Table **2**).

All cohort studies were assessed to have an moderate risk of bias, while the two randomized clinical trial subanalyses were deemed to have a low risk of bias (Table **3**) (for more details, see Supplementary Material - Tables **S4** and **S5**).

### 3.3 Outcomes

**Fig. (2):**
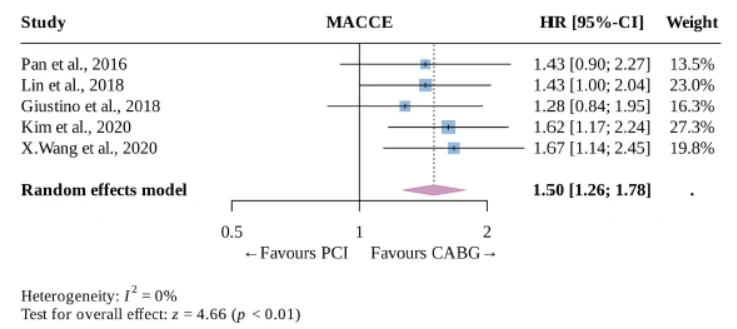
Forest plot comparing long-term MACCE in patients undergoing PCI *versus* CABG. **Abbreviations:** HR, hazard ratio; CI, confidence interval; MACCE, major adverse cardiac and cerebrovascular events; PCI, percutaneous coronary intervention; CABG, coronary artery bypass grafting.

**Fig. (3):**
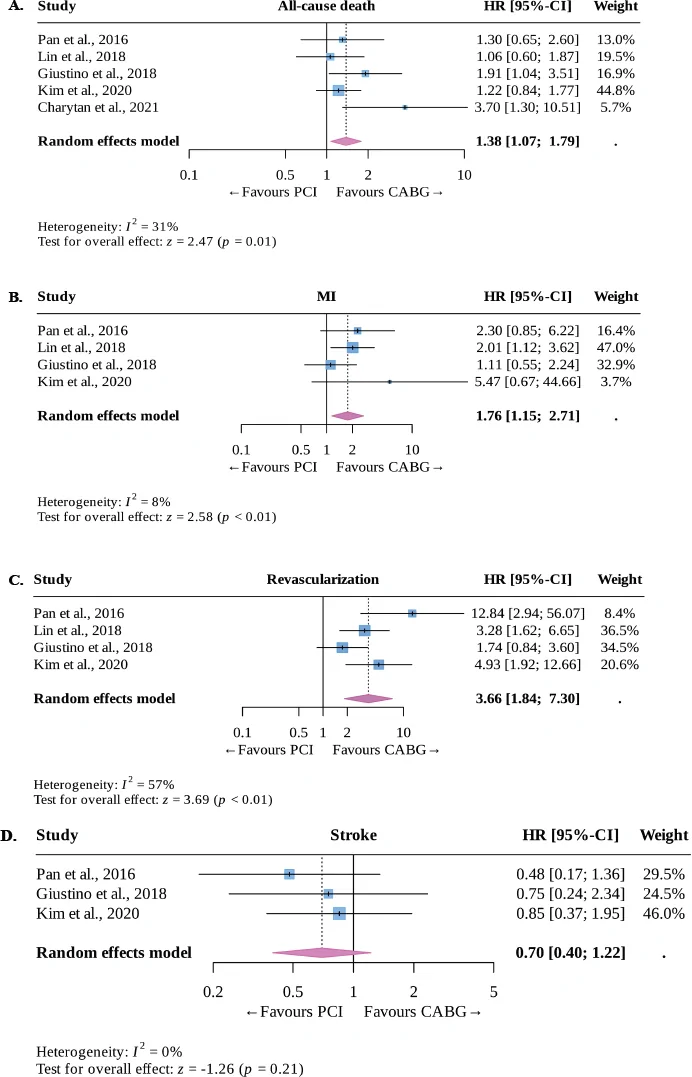
Forest plots comparing the incidence of individual adverse clinical outcomes in patients with LMCAD and CKD treated with PCI *versus* CABG. Panel (**A**) shows the hazard ratio for all-cause mortality; Panel (**B**) shows the hazard ratio for MI; Panel (**C**) shows the hazard ratio for ischemia-driven revascularization; and Panel (**D**) shows the hazard ratio for stroke. **Abbreviations:** HR, hazard ratio; CI, confidence interval; MI, myocardial infarction; PCI, percutaneous coronary intervention; CABG, coronary artery bypass grafting.

The pooled analysis of data demonstrated that patients treated with PCI had a significantly higher incidence of MACCE compared to those who underwent CABG (pooled hazard ratio [HR]: 1.50, 95% confidence interval [CI]: 1.26-1.78, *p<* 0.01, I^2^=0%) (Fig. **2**). Further analysis of the individual components of MACCE revealed that this increased risk was driven by a higher incidence of all-cause mortality (HR: 1.38, 95% CI: 1.07-1.79, *p=* 0.01, I^2^ = 31%) (Fig. **3A**), MI (HR: 1.76, 95% CI: 1.15-2.71, *p<* 0.01, I^2^ = 8%) (Fig. **3B**), and ischemia-driven revascularization (HR: 3.66, 95% CI: 1.84-7.30, *p<* 0.001, I^2^ = 57%) (Fig. **3C**). In contrast, no significant difference in the incidence of stroke was observed between the two procedures (HR: 0.70, 95% CI: 0.40-1.22, *p=* 0.21, I^2^ = 0%) (Fig. **3D**). Additionally, there was no significant difference in 30-day all-cause mortality between the two procedures (risk ratio [RR]: 1.28; 95% CI: 0.85-1.94; *p*=0.86; I^2^=57%).

For cardiovascular death, 3-point MACCE, and acute kidney injury, data were provided in less than three studies; thus, no meta-analysis was conducted. For cardiovascular death, Pan *et al*. [21] found no significant difference between PCI and CABG (7.5% *vs.* 4.5%; *p*=0.110), a finding supported by Giustino *et al*. (8% *vs.* 6%; *p*=0.610) [24]. Regarding three-point MACCE, Giustino *et al*. reported no significant difference between PCI and CABG (23.1% *vs.* 18.4%; *p*=0.331) [24], consistent with Milojevic *et al*. (17.7% *vs.* 15.3%; *p*=0.330) [22]. Furthermore, Giustino *et al*. was the only study to address acute kidney injury, showing a lower incidence in the PCI group compared to the CABG group (4% *vs.* 14%; *p*=0.034) [24].

### 3.4 Secondary Analyses of Outcomes

Subgroup analyses showed no statistically significant differences between subgroups when stratified by CKD stage, study type, age (cutoff at 70 years), or risk of bias assessment. Notably, however, for the primary outcome of MACCE, patients with CKD stage 3A (GFR 60-45 mL/min/1.73 m^2^) had a HR of 1.26 (95% CI: 0.84-1.87), whereas those with more advanced CKD (stage ≥ 4, GFR < 30 mL/min/1.73 m^2^) had a higher HR of 2.05 (95% CI: >1.31-3.20), although the subgroup difference was not statistically significant (*p =* 0.1099; see Supplementary Figure **S1**).

For the 30-day all-cause mortality outcome, the high level of heterogeneity (I^2^=57%) was largely attributable to the study conducted by Charytan *et al*., which favored CABG [27]. Sensitivity analysis identified this study as an outlier. Therefore, excluding this study from the meta-analysis resulted in no significant difference between the procedures (HR: 0.48; 95% CI: 0.17-1.34; *p*<0.001; I^2^=0%).

Finally, no publication bias was detected for MACCE based on the assessment of funnel plots and Egger’s tests (see Supplementary Fig. **S2**).

## 4 DISCUSSION

In this pooled analysis of 3,376 patients with LMCAD and CKD, the short- and long-term outcomes of PCI *versus* CABG were compared. Patients who underwent PCI had a higher incidence of MACCE, primarily attributable to a higher risk of all-cause mortality, MI, and ischemia-driven revascularization. In contrast, there were no differences in stroke and 30-day all-cause death. However, there were not enough data for an analysis of cardiovascular death, 3-point MACCE, and acute kidney injury events.

For the primary outcome of MACCE, our results are consistent with the EXCEL and NOBLE trials but contrast with the PRECOMBAT and the LMCAD subset of the SYNTAX trials [4-7]. The EXCEL trial, which included patients with LMCAD of low to moderate anatomical complexity, reported no significant difference in 3-point MACCE, a composite of death, stroke, or MI, between PCI and CABG (22% *vs.* 19.2%, *p*=0.11) [6]. However, when ischemia-driven revascularization was included in the primary endpoint, CABG demonstrated a clear advantage. Similarly, the NOBLE trial, which compared PCI with second-generation drug-eluting stents *versus* CABG in 1,201 patients with unprotected LMCAD, reported significantly higher MACCE rates in the PCI group (28% *vs.* 18%, *p*=0.004) [7]. In contrast, the PRECOMBAT trial showed no significant difference in overall MACCE between PCI and CABG in 600 patients with LMCAD (17.5% *vs.* 14.3%, *p*=0.26), though revascularization rates were higher in the PCI group (11.4% *vs.* 5.5%, *p=*0.012) [4]. Finally, the LMCAD subset of the SYNTAX trial, which compared PCI with first-generation (paclitaxel) drug-eluting stents to CABG in 705 patients with LMCAD and/or MVD, reported no significant difference in MACCE rate (36.9% *vs.* 31.0%, *p*=0.12) with an increased need for repeat revascularization [5]. It is important to note that patients with CKD were underrepresented from many of these trials, due to their elevated procedural risk, higher comorbidity burden, and increased baseline mortality. These factors posed both ethical and methodological challenges for randomization and outcome interpretation, limiting the applicability of these trial findings to the CKD population.

Our analysis revealed a higher incidence of MACCE in patients with LMCAD and CKD treated with PCI. This elevated MACCE rate was driven not only by an increase in ischemia-driven revascularization, as seen in the EXCEL and NOBLE trials, but also by a greater incidence of all-cause death and MI. Notably, the EXCEL trial was the only clinical trial that showed higher incidence of both all-cause mortality and MI in the PCI group. Similarly, FREEDOM trial, which enrolled diabetic patients with multivessel coronary artery disease reported similar outcomes [32]. It is plausible that the adverse outcomes observed in our study may be attributable either to the presence of diabetic nephropathy in many diabetic patients or to the independent effects of CKD on these outcomes, potentially to a similar extent as diabetes mellitus. These findings suggest that CKD may play a role in revascularization decision-making as significant as diabetes. Finally, due to limited data, a reliable analysis of MACCE rates in PCI across different CKD stages was not possible, but a trend toward increased risk with advancing CKD severity compared to CABG was observed.

The higher mortality in the PCI group in our analysis can be largely attributed to two key studies: the Giustino *et al*. subgroup analysis within the EXCEL trial and the Charytan *et al*. cohort study [24, 27]. In Giustino *et al*.'s study, the increased mortality in the PCI group was mainly due to non-cardiac causes, particularly sepsis occurring more than a month after the procedure (5.4% *vs.* 1.1%, *p=* 0.02). This finding was unexpected, as CABG is generally associated with a higher risk of infection. In the Charytan *et al*. study, the survival disadvantage associated with PCI was primarily driven by the presence or absence of prior myocardial infarction [27]. As for short-term mortality, there was no significant difference in 30-day all-cause death between PCI and CABG, likely due to a balance between early perioperative surgical risks in CABG and the risk of early stent thrombosis following PCI, especially in patients with CKD [33].

The higher incidence of MI and ischemia-driven revascularization observed in the PCI group may be attributed to several pathophysiological mechanisms that impair vascular healing and promote stent failure in patients with CKD. CKD is associated with complex hemostatic abnormalities, reduced efficacy of antiplatelet therapy, and diffuse atherosclerosis [34-37]. Moreover, chronic systemic inflammation in CKD promotes vascular smooth muscle cell proliferation, leading to neointimal hyperplasia and in-stent restenosis. Together, these processes compromise stent integrity and likely account for the increased rates of MI and repeat revascularization observed in our meta-analysis. The heterogeneity identified in our analysis may be partly explained by the limited number of studies included, the variability in CKD severity across studies, ranging from stage 3A to end-stage renal disease, as well as differences in study design and variations in procedural techniques and stent technologies. However, the absence of sufficiently detailed data precluded stratified analyses based on CKD stage, making future studies incorporating CKD stage-specific stratification essential.

Finally, no difference was observed in stroke rates between the two treatment strategies. However, a non-significant trend favoring PCI was noted, likely due to the higher periprocedural stroke risk associated with CABG from aortic manipulations and on-pump bypass use. The increasing use of off-pump bypass techniques with minimal aortic manipulation (as employed in CABG patients in the EXCEL trial) and careful peri-procedural evaluation and preparation may further reduce stroke risk for these patients [6].

The heterogeneity observed in our analysis, particularly for ischemia-driven revascularization (I^2^=57%), likely stems from several key factors. First, variability in CKD severity across studies, ranging from stage 3A to end-stage renal disease, may have differentially impacted outcomes, as suggested by our subgroup analysis showing a tendency to worse PCI outcomes with advanced CKD. Second, differences in study designs introduced methodological variability, with RCT subanalyses typically employing stricter inclusion criteria that may not fully represent real-world CKD populations seen in observational studies. Third, evolving techniques across the study period, including the transition from first- to second-generation stents in PCI and off-pump approaches in CABG, introduced technological heterogeneity. Importantly, while these factors contributed to variability in effect sizes, the consistent direction of effect favoring CABG across all studies strengthens our primary conclusion. However, the non-significant interaction by CKD stage (*p*=0.11) suggests the need for future studies to explicitly examine whether PCI may remain a reasonable option for select patients with milder renal impairment.

The inferior long-term outcomes of PCI compared to CABG in patients with concomitant LMCAD and CKD carry significant clinical implications, particularly as this high-risk population is frequently excluded from major randomized trials and underrepresented in current revascularization guidelines. Given the progressive nature of CKD and its impact on vascular biology and procedural outcomes, these data support a more cautious approach to PCI in patients with advanced renal impairment, underscoring the importance of incorporating CKD stage into revascularization decisions. Moreover, advanced imaging techniques, such as speckle-tracking echocardiography, intravascular imaging, and the integration of sophisticated artificial intelligence, models may enhance risk stratification and improve clinical outcomes following PCI [38, 39]. Collectively, these findings may help inform future guideline updates by encouraging greater attention to renal function when selecting optimal revascularization strategies for patients with LMCAD and may trigger further research through well-designed CKD-focused randomized controlled trials for more robust evidence.

## CONCLUSION

This meta-analysis demonstrates that revascularization of patients with LMCAD and CKD with PCI is associated with higher long-term risks of MACCE, all-cause mortality, MI, and repeat revascularization compared to CABG, favoring the latter as the preferred revascularization strategy for this high-risk population. However, dedicated, well-powered randomized controlled trials focusing specifically on this LMCAD-CKD population are essential to refine revascularization guidelines and optimize clinical decision-making for these complex patients. These prospective CKD-focused trials should employ standardized definitions for LMCAD and CKD, incorporate contemporary PCI techniques, including intravascular imaging, utilize advanced surgical approaches, and stratify patients according to CKD stages to ensure precise risk assessment and tailored therapeutic strategies.

## STUDY LIMITATIONS

It is important to acknowledge the limitations of this analysis. First, most of the included studies were observational, which may limit internal validity due to potential confounding by indication, as treatment selection (PCI *vs.* CABG) inherently reflected clinicians' assessments of unmeasured patient- and lesion-specific risk factors. Second, there was heterogeneity in PCI and surgical techniques, as well as in outcome definitions, across studies. Third, variability in CKD severity among patients suggests a need for further data to assess risk across different CKD stages. Finally, data on procedure-related safety outcomes, including acute kidney injury and 3-point MACCE, were insufficient for analysis. Despite these limitations, our systematic review and meta-analysis provides meaningful insights into the optimal interventional treatment for patients with both LMCAD and CKD.

## Figures and Tables

**Table 1:** Baseline characteristics of included studies.

**Study Type**	**Population Origin**	**Follow-up Time (Years)**	**No. of Total Patients**	**PCI Patients**	**CABG Patients**	**Age (Years)**	**Male** **Gender, n (%)**	**LVEF (%), Mean ± SD**	**eGFR (ml/min/** **1.73 m^2^), Mean ± SD**	**Hemodialysis, n (%)**	**References**
-	-	-	-	-	-	PCI / CABG	PCI / CABG	PCI / CABG	PCI / CABG	PCI / CABG	-
Retrospective cohort study	China	2.6±1.3	353	147	206	69 ± 6/ 68±6	108 (73.5) / 155 (75.2)	62±12 / 57±12	-	-	Pan *et al*., 2016 [21]
RCT subanalysis	17 countries across the USA and Europe (SYNTAX trial subanalysis)	5	127	67	60	-	-	-	-	-	Milojevic *et al*., 2018 [22]
Cohort study	Taiwan	3.5 (1.5-5.9)	185	84	101	74±10 / 73±10	72 (85.7) / 83 (82.2)	45±12 / 47±12	36±19 / 36±18	17 (20) / 16 (16)	Lin *et al*., 2018 [23]
RCT subanalysis	USA (EXCEL trial subanalysis)	3	361	177	184	73±8*	-	55±11*	49 ± 10 *	-	Giustino *et al*., 2018 [24]
Cohort study	Korea, Asia-Pacific (IRIS-MAIN registry)	3.5 (2-5.2)	891	536	355	70±9 / 68±8	398 (74.3) / 268 (75.5)	CHF: 33 patients / CHF: 27 patients	-	64 (12) / 37 (10)	Kim *et al*., 2020 [25]
Cohort study	China	Up to 3	368	161	207	68±8 / 68±8	121 (75.2) / 159 (76.8)	LVEF<50%: 61 (37.9%) / LVEF<50%: 74 (35.7%)	48±17 / 47±19	24 (15) / 36 (17)	X. Wang *et al*., 2020 [26]
Cohort study	Massachusetts, USA	5.4 (3.1-7.1)	1,091	462	629	-	-	-	-	-	Charytan *et al*., 2021 [27]
-	-	-	3,376	1,634	1,742	70.4 / 68.6	75.3% / 76.5%	**	**	**	Total / Weighted Mean

**Note:** Values are presented as mean ± SD or as n (%).

*Mean of both groups.

**Due to variability in reporting (*e.g.*, LVEF, eGFR *vs.* creatinine, presence of CKD *vs.* hemodialysis), the data are presented as reported in the original studies.

***Stage 4 CKD, not specified if on dialysis.

**Abbreviations:** PCI, percutaneous coronary interventions; CABG, coronary artery bypass grafting; SD, standard deviation; LVEF, left ventricle ejection fraction; CHF, congestive heart failure; eGFR, estimated glomerular filtration rate.

**Table 2:** Patients' baseline extent of vessel lesions.

**Extent of Diseased Vessels**	**PCI**	**CABG**	***p*-value**	**References**
MVD, n (%)	59 (74)	119 (97)	MVD: <0.001	Pan *et al*., 2016 [21]
CTO, n (%)	14 (18)	50 (41)	CTO: 0.001	-
LM only, n (%)	2 (2)	3 (3)	0.010	Lin *et al*., 2018 [23]
LM + 1-vessel disease, n (%)	11 (13)	6 (6)	-	-
LM + 2-vessel disease, n (%)	26 (31)	16 (16)	-	-
LM + 3-vessel disease, n (%)	45 (54)	76 (75)	-	-
Low or intermediate anatomical complexity (SYNTAX score <32)	Data on SYNTAX score reported, but no breakdown by extent of diseased vessels	-	-	Giustino *et al*., 2018 [24]
LM only, n (%)	6 (4)	3 (1)	0.024	Kim *et al*., 2020 [25]
LM + 1-vessel disease, n (%)	14 (9)	14 (7)	-	-
LM + 2-vessel disease, n (%)	41 (26)	39 (19)	-	-
LM + 3-vessel disease, n (%)	100 (62)	151 (73)	-	-

**Note:** Values are presented as n (%). **Abbreviations:** PCI, percutaneous coronary intervention; CABG, coronary artery bypass grafting; MVD, multivessel disease; CTO, chronic total occlusion; LM, left main artery.

The studies by Milojevic *et al.*, X. Wang *et al.*, and Charytan *et al.* did not include data on the extent of diseased vessels.

**Table 3:** Summary of risk of bias assessment.

**Study Design**	**Overall Risk of Bias**	**Assessment Tool**	**References**
Observational	Moderate	ROBINS-I	Pan *et al*., 2016 [21]
Observational	Moderate	ROBINS-I	Lin *et al*., 2018 [23]
Observational	Moderate	ROBINS-I	Kim *et al*., 2020 [25]
Observational	Moderate	ROBINS-I	X. Wang *et al*., 2020 [26]
Observational	Moderate	ROBINS-I	Charytan *et al*., 2021 [27]
RCT Subanalysis	Low	RoB-2	Milojevic *et al*., 2018 [22]
RCT Subanalysis	Low	RoB-2	Giustino *et al*., 2018 [24]

## Data Availability

All data generated or analyzed during this study are included in this published article.

## LIST OF ABBREVIATIONS

CABG: Coronary Artery Bypass Grafting
CHF: Congestive Heart Failure
CKD: Chronic Kidney Disease
GFR: Glomerular Filtration Rate
KDIGO: Kidney Disease Improving Global Outcomes
LMCAD: Left Main Coronary Artery Disease
LVEF: Left Ventricular Ejection Fraction
MACCE: Major Adverse Cardiac and Cerebrovascular Events
MI: Myocardial Infarction
MVD: Multivessel Disease
PCI: Percutaneous Coronary Intervention
RCTs: Randomized Controlled Trials
RoB2: Risk of Bias 2 Tool for Randomized Trials
ROBINS-I: Risk of Bias In Non-randomized Studies

## STANDARDS OF REPORTING

PRISMA guidelines were followed.
