# Supplementary material for: Left Main Revascularization in Patients with Chronic Kidney Disease: A Systematic Review and Meta-Analysis
Source: Curr Cardiol Rev. 2025 Sep 17;22(4):E1573403X396917. doi: 10.2174/011573403X396917250910215747 (PMC13519571; doi:10.2174/011573403X396917250910215747)
Supplement: Supplementary file 1 [file CCR-22-4-E1573403X396917_SD1.pdf]

Supplementary Material

Left Main Revascularization in Patients with Chronic Kidney Disease: A Systematic Review and Meta-Analysis

Ioannis Gialamas[1,*#], Konstantinos Kalogeras[1,#], Panteleimon Pantelidis[1], Georgios E. Zakynthinos[1], Antonios Lysandrou[1], Efstratios Katsianos[1], Athina Goliopoulou[1], Maria Ioanna Gounaridi[1], Nikolaos Vythoulkas-Biotis[1], Ourania Katsarou[1], Evangelos Oikonomou[1], Gerasimos Siasos[1,2] and Manolis Vavuranakis[1]

[1]3rd Department of Cardiology, Sotiria Chest Disease Hospital, Medical School, National and Kapodistrian University of Athens, Athens, Greece; [2]Cardiovascular Division of Brigham and Women's Hospital, Harvard Medical School, Boston, USA

Section and Topic	Item #	Checklist item	Location where item is reported
**TITLE**			
Title	1	Identify the report as a systematic review.	Page 1
**ABSTRACT**			
Abstract	2	See the PRISMA 2020 for Abstracts checklist.	Page 2
**INTRODUCTION**			
Rationale	3	Describe the rationale for the review in the context of existing knowledge.	Page 4
Objectives	4	Provide an explicit statement of the objective(s) or question(s) the review addresses.	Page 4
**METHODS**			
Eligibility criteria	5	Specify the inclusion and exclusion criteria for the review and how studies were grouped for the syntheses.	Page 5
Information sources	6	Specify all databases, registers, websites, organisations, reference lists and other sources searched or consulted to identify studies. Specify the date when each source was last searched or consulted.	Page 5, Supplementary Appendix 3
Search strategy	7	Present the full search strategies for all databases, registers and websites, including any filters and limits used.	Page 5, Supplementary Appendix 3
Selection process	8	Specify the methods used to decide whether a study met the inclusion criteria of the review, including how many reviewers screened each record and each report retrieved, whether they worked independently, and if applicable, details of automation tools used in the process.	Page 5
Data collection process	9	Specify the methods used to collect data from reports, including how many reviewers collected data from each report, whether they worked independently, any processes for obtaining or confirming data from study investigators, and if applicable, details of automation tools used in the process.	Pages 6-7
Data items	10a	List and define all outcomes for which data were sought. Specify whether all results that were compatible with each outcome domain in each study were sought (e.g. for all measures, time points, analyses), and if not, the methods used to decide which results to collect.	Page 6, Supplementary Appendix 4
	10b	List and define all other variables for which data were sought (e.g. participant and intervention characteristics, funding sources). Describe any assumptions made about any missing or unclear information.	Page 6, Supplementary Appendix 4
Study risk of bias assessment	11	Specify the methods used to assess risk of bias in the included studies, including details of the tool(s) used, how many reviewers assessed each study and whether they worked independently, and if applicable, details of automation tools used in the process.	Page 6
Effect measures	12	Specify for each outcome the effect measure(s) (e.g. risk ratio, mean difference) used in the synthesis or presentation of results.	Page 7

Section and Topic	Item #	Checklist item	Location where item is reported
Synthesis methods	13a	Describe the processes used to decide which studies were eligible for each synthesis (e.g. tabulating the study intervention characteristics and comparing against the planned groups for each synthesis (item #5)).	Page 5
	13b	Describe any methods required to prepare the data for presentation or synthesis, such as handling of missing summary statistics, or data conversions.	Pages 6-7
	13c	Describe any methods used to tabulate or visually display results of individual studies and syntheses.	Page 7
	13d	Describe any methods used to synthesize results and provide a rationale for the choice(s). If meta-analysis was performed, describe the model(s), method(s) to identify the presence and extent of statistical heterogeneity, and software package(s) used.	Page 7
	13e	Describe any methods used to explore possible causes of heterogeneity among study results (e.g. subgroup analysis, meta-regression).	Page 7
	13f	Describe any sensitivity analyses conducted to assess robustness of the synthesized results.	Page 7
Reporting bias assessment	14	Describe any methods used to assess risk of bias due to missing results in a synthesis (arising from reporting biases).	Page 7
Certainty assessment	15	Describe any methods used to assess certainty (or confidence) in the body of evidence for an outcome.	Page 7
**RESULTS**			
Study selection	16a	Describe the results of the search and selection process, from the number of records identified in the search to the number of studies included in the review, ideally using a flow diagram.	Pages 7-8, Figure 1
	16b	Cite studies that might appear to meet the inclusion criteria, but which were excluded, and explain why they were excluded.	Supplementary Appendix 5
Study characteristics	17	Cite each included study and present its characteristics.	Page 8, Tables 1-2
Risk of bias in studies	18	Present assessments of risk of bias for each included study.	Page 8, Supplementary Table S4 and S5
Results of individual studies	19	For all outcomes, present, for each study: (a) summary statistics for each group (where appropriate) and (b) an effect estimate and its precision (e.g. confidence/credible interval), ideally using structured tables or plots.	Pages 9-10, Figures 2-3
Results of syntheses	20a	For each synthesis, briefly summarise the characteristics and risk of bias among contributing studies.	Pages 9-10
	20b	Present results of all statistical syntheses conducted. If meta-analysis was done, present for each the summary estimate and its precision (e.g. confidence/credible interval) and measures of statistical heterogeneity. If comparing groups, describe the direction of the effect.	Pages 9-10, Figures 2-3
	20c	Present results of all investigations of possible causes of heterogeneity among study results.	Pages 9-10,
	20d	Present results of all sensitivity analyses conducted to assess the robustness of the synthesized results.	Pages 9-10,
Reporting biases	21	Present assessments of risk of bias due to missing results (arising from reporting biases) for each synthesis assessed.	Pages 9-10
Certainty of evidence	22	Present assessments of certainty (or confidence) in the body of evidence for each outcome assessed.	Pages 9-10
**DISCUSSION**			
Discussion	23a	Provide a general interpretation of the results in the context of other evidence.	Pages 10-11
	23b	Discuss any limitations of the evidence included in the review.	Pages 12-13
	23c	Discuss any limitations of the review processes used.	Pages 12-13
	23d	Discuss implications of the results for practice, policy, and future research.	Pages 15
**OTHER INFORMATION**			
Registration and protocol	24a	Provide registration information for the review, including register name and registration number, or state that the review was not registered.	Page 4
	24b	Indicate where the review protocol can be accessed, or state that a protocol was not prepared.	Page 4
	24c	Describe and explain any amendments to information provided at registration or in the proto-	Page 4

Section and Topic	Item #	Checklist item	Location where item is reported
		col.	
Support	25	Describe sources of financial or non-financial support for the review, and the role of the funders or sponsors in the review.	Page 14
Competing interests	26	Declare any competing interests of review authors.	Page 14
Availability of data, code and other materials	27	Report which of the following are publicly available and where they can be found: template data collection forms; data extracted from included studies; data used for all analyses; analytic code; any other materials used in the review.	Online supplementary data

From: Page MJ, McKenzie JE, Bossuyt PM, Boutron I, Hoffmann TC, Mulrow CD, et al. The PRISMA 2020 statement: an updated guideline for reporting systematic reviews. BMJ 2021;372:n71. doi: 10.1136/bmj.n71

For more information, visit: http://www.prisma-statement.org/

SUPPLEMENTARY MATERIAL - LITERATURE SEARCH

Two independent researchers (IG and PP) conducted a systematic search across three major databases—MEDLINE (via PubMed), Embase, and CENTRAL (via Cochrane)-from their inception through February, 2025. The search strategy incorporated alternative spellings, truncations, and variations of terms related to "percutaneous coronary intervention", "coronary artery bypass graft surgery", "left main coronary artery disease", and "chronic kidney disease", which are listed in more details below. To enhance the completeness of the search, we also implemented a snowballing technique by manually screening the reference lists of all included articles and relevant reviews. The search results were imported into a reference management software (Zotero) for deduplication, followed by screening through a systematic review management platform (https://sr-accelerator.com/) to facilitate blinded and independent title/abstract and full-text screening. The search was performed without date or language restrictions.

VARIATIONS OF IMPORTANT SEACH TERMS:

Left main coronary disease

[1] "Coronary Artery Disease"[Mesh]

[2] “left main coronary artery disease”

[3] “left main coronary disease”

[4] “left main disease*”

[5] LMCA*

[6] [1] OR [2] OR [3] OR [4] OR [5]

Percutaneous coronary intervention

[7] "Percutaneous Coronary Intervention"[Mesh]

[8] “percutaneous coronary intervention”

[9] PCI

[10] PTCA

[11] angioplasty

[12] stent*

[13] atherectomy

[14] [7] OR [8] OR [9] OR [10] OR [11] OR [12] OR [13]

Coronary artery bypass grafting

[15] "Coronary Artery Bypass"[Mesh]

[16] “coronary artery bypass”

[17] bypass

[18] surg*

[19] CABG

[20] [15] OR [16] OR [17] OR [18] OR [19]

Chronic kidney disease

[21] "Renal Insufficiency, Chronic"[Mesh]

[22] “renal insufficiency”

[23] “kidney insufficiency”

[24] “kidney disease*”

[25] “renal disease*”

[26] CKD

[27] “renal dysfunction”

[28] “kidney dysfunction”

[29] nephropathy

[30] “kidney failure”

[31] “renal failure”

[32] [21] OR [22] OR [23] OR [24] OR [25] OR [26] OR [26] OR [27] OR [28] OR [29] OR [30] OR [31]

[6] AND [14] AND [20] AND [32]

PUBMED SEARCH STRATEGY:

("Coronary Artery Disease"[Mesh] OR "left main coronary artery disease" OR "left main coronary disease" OR "left main disease*" OR LMCA*) AND ("Percutaneous Coronary Intervention"[Mesh] OR "percutaneous coronary intervention" OR PCI OR PTCA OR angioplasty OR stent* OR atherectomy) AND ("Coronary Artery Bypass"[Mesh] OR "coronary artery bypass" OR bypass OR surg* OR CABG) AND ("Renal Insufficiency, Chronic"[Mesh] OR "renal insufficiency" OR "kidney insufficiency" OR "kidney disease*" OR "renal disease*" OR CKD OR "renal dysfunction" OR "kidney dysfunction" OR nephropathy OR "kidney failure" OR "renal failure")

EMBASE SEARCH STRATEGY:

('coronary artery disease'/exp OR 'left main coronary artery disease' OR 'left main coronary disease' OR 'left main disease*' OR LMCA*) AND ('percutaneous coronary intervention'/exp OR 'percutaneous coronary intervention' OR PCI OR PTCA OR angioplasty OR stent* OR atherectomy) AND ('coronary artery bypass graft'/exp OR 'coronary artery bypass' OR bypass OR surg* OR CABG) AND ('chronic kidney disease'/exp OR 'renal insufficiency' OR 'kidney insufficiency' OR 'kidney disease*' OR 'renal disease*' OR CKD OR 'renal dysfunction' OR 'kidney dysfunction' OR nephropathy OR 'kidney failure' OR 'renal failure')

CENTRAL SEARCH STRATEGY:

("left main coronary artery disease" OR "left main coronary disease" OR "left main disease*" OR LMCA*) AND ("percutaneous coronary intervention" OR PCI OR PTCA OR angioplasty OR stent* OR atherectomy) AND ("coronary artery bypass" OR bypass OR surg* OR CABG) AND ("chronic kidney disease" OR "renal insufficiency" OR "kidney insufficiency" OR "kidney disease*" OR "renal disease*" OR CKD OR "renal dysfunction" OR "kidney dysfunction" OR nephropathy OR "kidney failure" OR "renal failure")

SUPPLEMENTARY MATERIAL - ELIGIBILITY CRITERIA

Studies were considered eligible for inclusion if they met the following criteria based on PICO:

1. Population:

Patients with both

- LMCAD: Defined as the presence of greater than 50% stenosis in the left main coronary artery as determined by coronary angiography.

- CKD: Defined as patients with GFR less than 60 mL/min/1.73 m², regardless of the equation used to calculate, in accordance with the “KDIGO” guidelines.

2. Interventions and Comparators:

- Intervention Group: Patients who underwent PCI as the primary treatment procedure.
- Comparator Group: Patients who underwent CABG as the standard treatment for LMCAD.

3. Outcomes:

- Studies reported clinical outcomes comparing the efficacy and safety of PCI versus CABG.

4. Study Design:

- Both observational studies (prospective or retrospective cohort studies) and RCTs were eligible.
- Studies provided sufficient data to extract or calculate HRs, RRs, or ORs for the outcomes of interest.

5. Language and Publication Status:

- Only articles published in English language.
- Only studies published in peer-reviewed journals were considered, with full-text available.

SUPPLEMENTARY MATERIAL - DATA EXTRACTION

Data from eligible studies were extracted by two independent reviewers (I.G. and P.P.) using a predefined form in a spreadsheet, designed according to the Cochrane Checklist of Items. Any discrepancies were resolved through consensus.

The extracted data entailed study identification and design details, including author names, publication year, study type, cohort name, country of origin, enrollment period, follow-up duration, randomization method, and group sizes. Additionally, demographic and baseline characteristics such as age, sex, cardiovascular risk factors, LVEF, and extent of diseased vessels were collected. Each study's GFR equation and procedural specifics, such as the type and quantity of stents used in PCI, were also recorded.

SUPPLEMENTARY MATERIAL - OUTCOMES DEFINITIONS

Primary outcomes

- Major adverse cardiac and cerebrovascular events (MACCE): Composite of death from any cause, myocardial infarction, stroke, or any ischemia-driven revascularization procedure.
  - All-cause death: Death from any cause, including cardiovascular and non-cardiovascular causes.
  - Myocardial infarction (MI): The presence of myocardial injury with signs of ischemia such as chest pain, ECG changes, new wall motion abnormalities, or coronary occlusion on angiography.
  - Stroke: Loss of neurological function for at least 24 hours proven to be caused by an ischaemic or haemorrhagic event.
  - Revascularization: Any type of percutaneous or surgical revascularization procedure, irrespective of the target or non-target lesion.

Secondary outcomes

- Individual components of MACCE (all-cause death, MI, stroke, and revascularization).
- Cardiovascular death: Death from cardiovascular causes.
- 30-days all-cause death: Death from any cause, including non- and cardiovascular causes in the first 30 days.
- 3-point MACCE: Composite of death from any cause, myocardial infarction, or stroke.
- Events of acute kidney injury: Improving Global Outcome (KDIGO) definition:
  - Rise in serum creatinine ≥0.3 mg/dL within 48 hours.
  - Rise in serum creatinine ≥1.5 times baseline, which is known or presumed to have occurred within the prior seven days.
  - Urine output < 0.5 mL/kg/hour for six hours

Supplementary Material - Search results and study characteristics

Initially, a total of 6,059 titles were retrieved from three major literature databases after deduplication. Following title evaluation, 92 articles underwent abstract evaluation, and subsequently, 20 articles underwent a comprehensive assessment. Ultimately, 7 studies met the inclusion criteria and were included in the subsequent analysis [21–27].

The study by Milojevic et al., apart from patients with LMCAD, also included patients with multivessel coronary disease [22]. Nonetheless, 40% of total patients had LMCA, and 3-point MACCE was reported for this subgroup of patients. Therefore, we decided to include this study in the present systematic review and meta-analysis. An attempt to contact the corresponding author was made to acquire additional data, but no response was received. Moreover, the study by Charytan et al. included a mixed population comprising patients with and without left main involvement [27]. However, they also analyzed outcomes including all-cause death and new-onset end-stage renal failure. The previous author responded to our email, but it was not possible to provide us with more study data.

Among rejected articles, it is notable to mention the study by Li et al., despite meeting the inclusion criteria, it was excluded because it involved patients who had already participated in the study conducted by Pan et al. [21, 30]. Furthermore, the studies by Yamamoto et al. and Marui et al. examined patients from the CREDO-Kyoto PCI/CABG Registry but did not specify patients with both LMCAD and CKD [28, 30]. Additionally, the study by Daoulah et al. used a different definition for CKD, raising the GFR threshold to 90mL/min/1.73 m^2 [29]. Efforts were made to contact the corresponding contact of the "CREDO-Kyoto PCI/CABG Registry" and Daoulah et al. study; however, no response was received.

Supplementary Table S1 - Raw data for the MACCE and the individual components

MACCE						
**Author**	**Method of extraction**	**HR**	**CI1**	**CI2**	**Log HR**	**SE HR**
Pan et al.,	direct	1.43	0.90	2.25	0.3577	0.2362
Lin et al.	indirect	1.43			0.3577	0.1809
Giustino et el.	direct	1.28	0.84	1.97	0.2469	0.2149
Milojevic et al.						
Kim et al.	direct	1.62	1.17	2.24	0.4824	0.166
X.Wang et al.	direct	1.67	1.14	2.44	0.5128	0.1948
Charytan et al.						
**All-cause death**						
**Author**	**Method of extraction**	**HR**	**CI1**	**CI2**	**Log HR**	**SE HR**
Pan et al.,	direct	1.30	0.65	2.57	0.2624	0.3537
Lin et al.	indirect	1.06			0.0583	0.2886
Giustino et el.	direct	1.91	1.04	3.52	0.6471	0.3101
Milojevic et al.						
Kim et al.	direct	1.22	0.84	1.75	0.1989	0.1904
X.Wang et al.						
Charytan et al.	direct	3.70	1.30	10.50	1.3083	0.5337
**MI**						
**Author**	**Method of extraction**	**HR**	**CI1**	**CI2**	**Log HR**	**SE HR**
Pan et al.,	direct	2.30	0.85	6.24	0.8329	0.5079
Lin et al.	indirect	2.01			0.6981	0.3001
Giustino et el.	direct	1.11	0.55	2.24	0.1044	0.3583
Milojevic et al.						

Kim et al.	direct	5.47	0.67	44.50	1.6993	1.0713
X.Wang et al.						
Charytan et al.						
**Stroke**						
**Author**	**Method of extraction**	**HR**	**CI1**	**CI2**	**Log HR**	**SE HR**
Pan et al.,	direct	0.48	0.17	1.32	-0.734	0.5296
Lin et al.						
Giustino et el.	direct	0.75	0.24	2.36	-0.2877	0.5814
Milojevic et al.						
Kim et al.	direct	0.85	0.37	1.93	-0.1625	0.4244
X.Wang et al.						
Charytan et al.						
**Revascularization**						
**Author**	**Method of extraction**	**HR**	**CI1**	**CI2**	**Log HR**	**SE HR**
Pan et al.,	direct	12.84	2.94	56.01	2.5526	0.7521
Lin et al.	indirect	3.28			1.1878	0.361
Giustino et el.	direct	1.74	0.84	1.97	0.5539	0.3716
Milojevic et al.						
Kim et al.	direct	4.93	1.92	12.67	1.5953	0.4811
X.Wang et al.						
Charytan et al.						

Abbreviations: HR, Hazard ratio; CI, Confidence interval; MACCE, Major adverse cardiac and cerebrovascular events; MI, myocardial infarction

Supplementary Table S2 - Raw data for cardiovascular death, acute kidney injury, and 3-point MACCE (composite of all-cause death, MI, or stroke)

**Cardiovascular death**						
**Author**	**Method of extraction**	**HR**	**CI1**	**CI2**	**Log HR**	**SE HR**
Pan et al.,	direct	1.77	0.72	4.38	0.571	0.4589
Lin et al.						
Giustino et el.	direct	1.34	0.61	2.94	0.2927	0.4015
Milojevic et al.						
Kim et al.						
X.Wang et al.						
Charytan et al.						
**Acute kidney injury**						
**Author**	**Method of extraction**	**HR**	**CI1**	**CI2**	**Log HR**	**SE HR**
Pan et al.,						
Lin et al.						
Giustino et el.	direct	0.30	0.10	0.88		

Milojevic et al.						
Kim et al.						
X.Wang et al.						
Charytan et al.						
3-point MACCE (all-cause death, MI, or stroke)						
Author	Method of extraction	HR	CI1	CI2	Log HR	SE HR
Pan et al.,						
Lin et al.						
Giustino et el.		1.25	0.79	1.98	0.2231	0.2341
Milojevic et al.		1.24	0.61	2.52	0.2151	0.3619
Kim et al.						
X.Wang et al.						
Charytan et al.						

Abbreviations: HR, Hazard ratio; CI, Confidence interval; MACCE, Major adverse cardiac and cerebrovascular events; MI, myocardial infarction

Supplementary Table S3 - Raw data for the 30-day MACCE and the individual components.

30-day MACCE					
Author		PCI total	CABG total	PCI events	CABG events
Pan et al.,					
Lin et al.		84	101	3	9
Giustino et el.		177	184	19	54
Milojevic et al.					
Kim et al.					
X.Wang et al.					
Charytan et al.					
30-day all-cause death					
Author		PCI total	CABG total	PCI events	CABG events
Pan et al.,					
Lin et al.		84	101	3	9
Giustino et el.		177	184	2	3
Milojevic et al.					
Kim et al.					
X.Wang et al.					
Charytan et al.		181	365	15	11
30-day MI					
Author		PCI total	CABG total	PCI events	CABG events
Pan et al.,					
Lin et al.		84	101	1	0

Giustino et el.		177	184	6	22
Milojevic et al.					
Kim et al.					
X.Wang et al.					
Charytan et al.					
30-day Stroke					
Author		PCI total	CABG total	PCI events	CABG events
Pan et al.,					
Lin et al.		84	101	0	1
Giustino et el.		177	184	2	3
Milojevic et al.					
Kim et al.					
X.Wang et al.					
Charytan et al.					
30-day revascularization					
Author		PCI total	CABG total	PCI events	CABG events
Pan et al.,					
Lin et al.		84	101	0	0
Giustino et el.		177	184	2	4
Milojevic et al.					
Kim et al.					
X.Wang et al.					
Charytan et al.					

Abbreviations: MACCE, Major adverse cardiac and cerebrovascular events; MI, myocardial infarction; PCI, percutaneous coronary intervention; CABG, coronary arterial bypass grafting

Supplementary Table S4 - Risk of bias assessment for observational studies.

Study, year of publication	Confounding	Selection Bias	Classification Bias	Deviations Bias	Missing Data	Outcome Measurement	Reporting Bias	
Pan et al., 2016	Moderate risk of bias	Moderate risk of bias	Moderate risk of bias	Low risk of bias	Low risk of bias	Low risk of bias	Low risk of bias	ROBINS-1
Lin et al., 2018	Moderate risk of bias	Moderate risk of bias	Moderate risk of bias	Low risk of bias	Low risk of bias	Low risk of bias	High risk of bias	
Kim et al., 2020	Moderate risk of bias	Moderate risk of bias	Moderate risk of bias	Low risk of bias	Low risk of bias	Low risk of bias	Low risk of bias	
X.Wang et al., 2020	Moderate risk of bias	Moderate risk of bias	Moderate risk of bias	Low risk of bias	Low risk of bias	Low risk of bias	High risk of bias	
Charytan et al., 2021	Moderate risk of bias	Moderate risk of bias	Moderate risk of bias	Low risk of bias	Low risk of bias	Low risk of bias	High risk of bias	

Supplementary Table S5. Risk of bias assessment for randomised control trial subanalyses

Study, year of publication	Randomization Process	Deviations from Interventions	Missing Outcome Data	Measurement of Outcome	Selection of Reported Result	
Giustino et al., 2018	Low risk of bias	Low risk of bias	Low risk of bias	Low risk of bias	Low risk of bias	RoB-2
Milojevic et al., 2018	Low risk of bias	Low risk of bias	Low risk of bias	Low risk of bias	Low risk of bias	

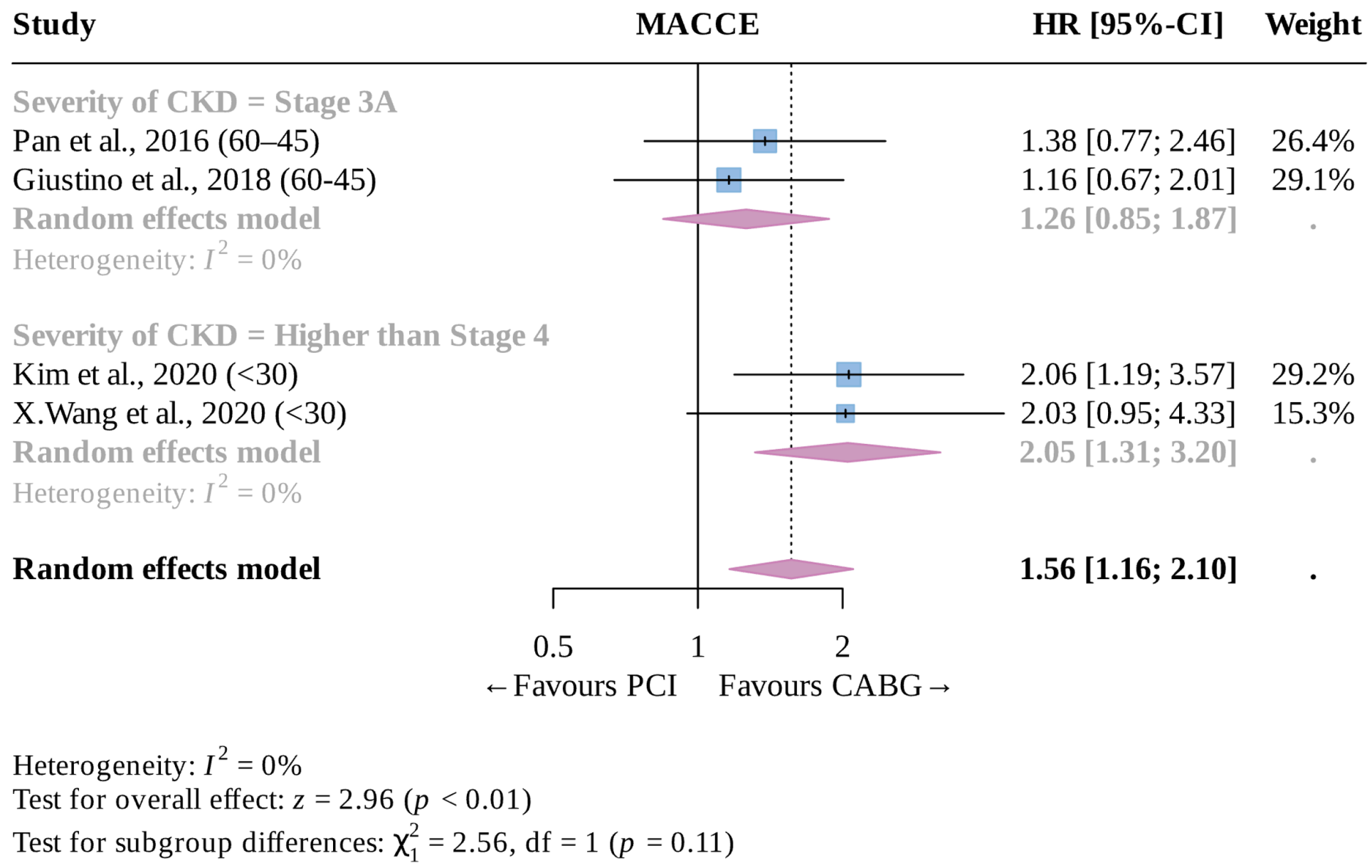

Supplementary Figure S1 – Subgroup analysis of MACCE based on CKD stages

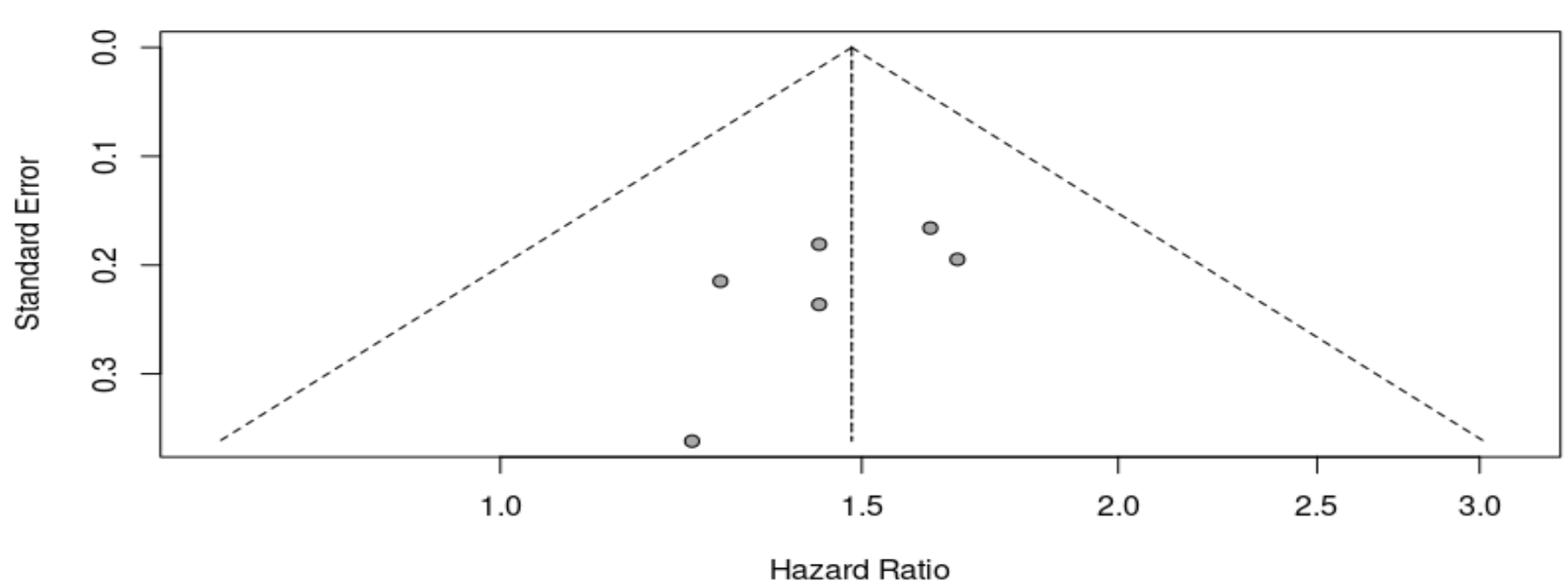

Supplementary Figure S2 - Publication bias funnel plot for the primary outcome of MACCE

In this case, we noticed a low Hedges' g, indicating that there is low publication bias. Studies are published regardless of their sample size or statistical significance. Therefore, the funnel plot appears symmetrical, with studies evenly distributed on both sides, indicating that both small and large studies with varying effect sizes for MACCE are equally likely to be published.

Abbreviation: MACCE, Major adverse cardiac and cerebrovascular event
